# Artificial Intelligence Applications in the Diagnosis of Neuromuscular Diseases: A Narrative Review

**DOI:** 10.7759/cureus.48458

**Published:** 2023-11-07

**Authors:** Martha C Piñeros-Fernández

**Affiliations:** 1 Pediatric Neurology, Fundación Cardio Infantil - La Cardio, Bogotá, COL; 2 Pediatric Neurology, Cobos Medical Center, Bogotá, COL

**Keywords:** diagnostic accuracy, applications of medical informatics, machine learning, artificial intelligence, neuromuscular diseases

## Abstract

The accurate diagnosis of neuromuscular diseases (NMD) is in many cases difficult; the starting point is the clinical approach based on the course of the disease and a careful physical examination of the patient. Electrodiagnostic tests, imaging, muscle biopsy, and genetics are fundamental complementary studies for the diagnosis of NMD. The large volume of data obtained from such studies makes it necessary to look for efficient solutions, such as artificial intelligence (AI) applications, which can help classify, synthesize, and organize the information of patients with NMD to facilitate their accurate and timely diagnosis. The objective of this study was to describe the usefulness of AI applications in the diagnosis of patients with neuromuscular diseases. A narrative review was done, including publications on artificial intelligence applied to the diagnostic methods of NMD currently existing. Twelve studies were included. Two of the studies focused on muscle ultrasound, five of the studies on muscle MRI, two studies on electromyography, two studies on amyotrophic lateral sclerosis (ALS) biomarkers, and one study on genes related to myopathies. The accuracy of classification using different classification algorithms used in each of the studies included in this narrative review was already 90% in most studies. In conclusion, the future design of more accurate algorithms applied to NMD with greater precision will have an impact on the earlier diagnosis of this group of diseases.

## Introduction and background

Neuromuscular diseases (NMDs) are a heterogeneous group of diseases that affect the muscle and the nerve, the neuromuscular junction, and the motor neuron. The etiology of NMD is variable, but in general, it can be classified in the NMD of genetic etiology [1], and the NMD of non-genetic etiology that occurs in infectious, autoimmune, inflammatory, paraneoplastic, and toxic processes [2].

Diagnosis of NMD

The diagnosis of NMD could be difficult and sometimes late, due to the variability of clinical manifestations. The initial diagnostic approach of all NMDs of both genetic and non-genetic causes is clinical, based on the symptoms presented by the patient, considering the age and the type of primary involvement of the peripheral nervous system suffered.

The process of diagnosing NMD requires complementary or confirmatory diagnostic tests of electrodiagnosis, imaging, and muscle biopsy to know the topographic or anatomical distribution of the disease, for example, which limbs or muscle groups present the greatest weakness, structural or functional damage, and confirmatory genetics testing in cases of suspected genetic NMD.

Artificial intelligence (AI)

The most basic definition of AI is the intelligence exhibited by machines, also described as "machines that mimic cognitive functions that are associated with the human mind related to learning and problem-solving" [3,4].

The European Commission AI High-Level Expert Group (AI HLEG) pointed out that AI refers to systems designed by humans, which given an objective can interact with the physical or digital world, by interpreting structured or unstructured data, and to reason based on the knowledge obtained from this data. In this way, its final objective is to present the best actions to take according to predefined parameters to achieve the initially proposed objective [5]. As a scientific discipline, AI is subdivided into domains and subdomains (Table 1) [5].

**Table 1:** Domains and subdomains of Artificial Intelligence

Reasoning
Knowledge representation
Automatic reasoning
Common sense reasoning
Planning
Planning and scheduling
Search
Optimization
Learning
Machine Learning
Communication
Natural Language Processing
Perception
Computer vision
Audio Processing
Integration and interaction
Multi-agent system
Robotics and automation
Connected and automated vehicles
Services
Artificial Intelligence Services
Ethics and philosophy
Ethics of artificial intelligence
Philosophy of artificial intelligence

Machine learning (ML)

ML refers to a mathematical model that can improve performance on a task when exposed to data. To design ML models, algorithms are necessary, which are the sequences of instructions that a computer system must comply with to achieve the resolution of a problem. According to the complexity of the model, the strategies and functions applied vary. There are four basic methods of machine learning. The first is supervised machine learning, the second is unsupervised learning, the third is semi-supervised learning, and the last is reinforcement machine learning [6].

In supervised machine learning, the model is provided with the correct answer in advance, therefore, it knows the question and knows the answer, that is, patterns defined between questions and answers. The process of developing a supervised ML model consists of training the algorithm, for which it is necessary to have three sets of data. The first is the training dataset from which the algorithm learns the optimal parameters to accomplish the task. The second is the test set, which is a dataset on which the performance of a parameterized model is based. The third group of data is the validation set, used to evaluate model performance, which differs from the test set used during training to establish hyperparameters or aspects of the model architecture that can be modified to optimize model performance [6].

The methods used to develop a supervised ML model are of two types: regression and classification. Regression methods include linear regression, generalized linear models, penalized regression models, regression trees, and vector support regression. The classification methods use Bayesian models, vector support (SVM), k nearest neighbors, and neural networks [7].

Unsupervised machine learning usually involves the analysis of unlabeled data, under assumptions about the structural properties of the data. The data can be algebraic, combinatorial, or probabilistic. The different assumptions are established by methods of dimension reduction, factor analysis, random projections, and automatic encoders or by statistical principles such as moments or Bayesian models [6]. The methods used to make unsupervised ML models are combination algorithms, self-organizing maps, and hierarchical clustering methods [7]. The semi-supervised ML model uses labeled and unlabeled data. It aims to provide a better result for prediction. Application areas of semi-supervised models among others, are machine translation, fraud detection, data labeling, and text classification [8].

Machine learning by reinforcement is based on the model’s ability to take actions according to patterns that are neither explicit nor predetermined, with cost and reward functions. In health care, a booster ML model would function as an agent between the medical device, the computer equipment, and the system. The model takes a particular action in a medical setting to elicit a specific reward and then uses evaluative feedback to improve its performance. Reinforcement ML applied to clinical decision-making tools of treatments requires frequent modifications for medical diagnosis to indicate the treatment, indicate alerts of the treatment, or question the human decision if it does not fit the interactions expected by the model [9].

Artificial neural network (ANN)

The ANN is a calculation algorithm that requires training. It is an analogy of the functioning of biological neurons and emulates the learning capacity of the nervous system. A neural network (NN) consists of multiple simple processors or connected nodes called neurons. Each node or neuron represents the output of another neuron with an assigned value according to the degree of interconnection, thus obtaining the weighting of the input, this is necessary for the training of the algorithm. The NN is composed of three layers or levels of nodes called neuronal layers, which resemble the layers of the cerebral cortex. The first layer is the input one that contains the neurons with the data from which the model will obtain the results proposed to hand. The second is the hidden layer, also called the “black box”, formed in turn by one or more layers not visible or observable. In multilayer models, the output layer will correspond to the input of the next layer, and the third layer is the output or result. The connections between neurons can be forward, backward, or lateral [10].

Deep learning (DL)

Deep learning Implies supervised and unsupervised ML techniques. This is what happens when an NN starts data processing and obtains basic knowledge. Besides understanding the data, it also learns from it, that is, the model delves into what it learns by itself without following a list of predetermined tasks. The input layer connects forward with the hidden layer; the interaction of the data is not visible or known and will be iterative depending on the number of layers created in the model; and to the right of the model is the layer of output neurons corresponding to the result. In an applied model, the connections between neurons are not exactly one-to-one [11].

Convolutional neural networks (CNN)

Convolutional networks are a type of neural network that uses a linear function called convolution. The purpose of convolution is to extract a limited number of features from each layer, so a filter slides over the input to produce a rectified activation map for feature identification. The next layer relates to that limited number of features, and the function repeats depending on the number of convolutional layers of the model until it reaches the output layer [12]. The kernel function is the most frequently used application in the elaboration of CNN models and consists of the conversion of a space of few dimensions into a space of greater dimension through complex operations of the data. It is a function that quantifies the similarity between two observations in a new dimensional space [13].

Machine learning in medicine

Machine learning technology allows doctors and researchers to predict which treatment and prevention strategies more accurately would be effective for a particular disease and in which groups of people those strategies will work. It requires sufficient computing power and algorithms that can learn by themselves at a high speed (e.g., deep learning) [14]. In the clinical field of practice, informatic tools such as Clinical Decision Support Systems (CDSS) aim to improve medical care by supporting clinical decisions and comparing a patient’s characteristics with a computerized database of clinical knowledge. The output data, in this case, are suggestions about specific evaluations or recommendations presented to the physician or patient for decision-making [15].

One of the applications of AI in ML-based image preprocessing via CNN is segmentation. It consists of delimiting the structure of the organ under study following the anatomical limits for each layer of the region of interest (ROI) done by technicians in the anatomical tracing using specific software tools. MRI can have hundreds of layers depending on the resolution of the study images, and therefore, manual segmentation is a slow, subjective, and laborious process [16]. The analysis of textures in muscle images made by AI allows the monitoring of NMD performing first- and second-order statistical tests. First-order statistical tests evaluate the distribution of gray-level frequencies of the pixel intensity histogram in a region of interest. Second-order statistical tests can be based on a co-occurrence matrix and include entropy, energy, homogeneity, dissimilarity, and correlation. Higher-order statistical tests like contrast, roughness, and occupancy can be calculated using neighboring gray-tone difference matrices, which examine the location and relationships between three or more pixels [17].

It is important to note that machine learning techniques will always have a small error rate. This is why it is essential to evaluate the accuracy of the model by the percentage of correct answers obtained when applying it. The application of AI has ethical implications, not only in the health field. This is where the concept of explainability arises, also referred to as interpretability and/or transparency of the algorithms used - it is understood as a characteristic that allows a person to reconstruct why in a certain AI model the predictions presented have occurred [18].

The objective of this study was to describe the usefulness of AI applications in the diagnosis of patients with neuromuscular diseases.

## Review

This review included 12 studies on artificial intelligence applied to the diagnostic methods of NMD.

Muscle imaging studies

Of the seven studies about muscle features in NMD included in this review, two studies focused on muscle ultrasound [19,20], and five on muscle MRI [21-25].

AI in Muscle Ultrasound

Two of the studies analyzed the application of AI in ultrasound studies [19,20]. Marzola et al study focused on muscle segmentation for ultrasound study and applied DL models with multiple learning architectures for the evaluation of NMD. Specifically, they performed the calculation of the mean grayscale value for automatic cross-sectional area in muscle ultrasound images acquired at different anatomical sites. The dataset of Marzola's study included 3917 images of the acquired biceps brachii, tibialis anterior, and medial gastrocnemii of 1283 subjects (mean age 50 ± 21 years, 729 males). The reported accuracy was 0.91 ± 0.08 for training data, 0.89 ± 0.10 for validation data, and 0.88 ± 0.12 for test data. They concluded that the applied model allowed to segment with optimal precision of the cross-sectional area of the muscles evaluated by ultrasound [19].

In Nodera et al's study, they used classification algorithms to differentiate between inflammatory myopathies and myotonic dystrophy. In the study, they performed a texture analysis of muscle ultrasound images of patients with inflammatory myopathies. Of these, 11 patients had inclusion body myositis and 21 polymyositis/dermatomyositis, with 19 patients with myotonic dystrophy (MD) type 1. The classification was performed by the Weka program (University of Waikato, New Zealand) and the classifiers were: simple logistic, support vector machine (SVM), and random forest (RF). The texture study focused on the analysis of roughness with the algorithm of comparison with neighboring pixels. Average pixel values were similar in all three groups. However, they found significant differences for pixel standard deviation values, entropy histogram, gray-level length matrix, and gray-level non-uniformity level. The classification accuracy obtained was 76% [20].

AI in Muscle Magnetic Resonance

Five studies of AI application in muscle magnetic resonance are presented in Table 2.

**Table 2:** AI precision results in muscle MRI FF: Fat tissue Fraction; NMD: neuromuscular disease; AI: Artificial Intelligence ^a ^Felisaz et al study results provided: mean absolute errors ranging from 0.110 to 0.133 for FF and 0.068 to 0.115 for (MRI weight T2) wT2

Study	Algorithm	Predictor	Type of NMD	Precision (%)
Chen [21]	Segmentation	FF	Neuropathies	99
Yang [22]	Classification	FF Comparison between AI and radiologist reports	Dystrophinopathies	AI 91 Radiologist 84
Felisaz [23]	Classification	FF	Facioscapulohumeral dystrophy	ND^a^
Verdú-Díaz [24]	Classification	FF specific-pattern disease	Muscular dystrophies	AI 96
Verdú-Díaz [24]	Classification	FF Comparison between AI and radiologist reports	Muscular dystrophies	AI 91 Radiologist 63
Gadermayr [25]	Classification	FF	Myopathy	88

Chen et al implemented a three-dimensional artificial network for the segmentation of muscle MRI images for the prediction of fat tissue fraction (FF). The images of 24 subjects with NMD were compared with those of 19 healthy subjects. Model accuracy was assessed using pixel accuracy and Dice coefficient (DC) compared to manual methods. CD values ranged from 83% to 98% for the thigh muscles and from 63% to 96% for the calf muscles. The accuracy values were 98.9% in the thigh and 97.1% in the calf muscles [21].

Yang et al studied a collection of MRI images of 148 cases of dystrophinopathies and 284 controls with other muscle diseases. They studied 2536 images of a total of 432 cases included. The region of interest was in the muscles of the right thigh. They used a DL model with CNN. The accuracy of the results of three expert radiologists was compared with the result of the model. The accuracy of the model was 91%, while the accuracy results of the three expert radiologists with a 95% CI were 80%, 84% and 84%. They concluded that the performance of the applied DL model was comparable to that observed with experienced radiologists [22].

The Felisaz et al study focused on the determination of the fat tissue fraction (FF) in muscle MRI performing eight ML models: linear regression, ridge regression, Lasso regression, generalized additive model, regression tree, RF, k-nearest-neighbors (kNN), SVM, and mixed effect model. With them, they sought to predict water content in T2 (wT2) and the FF by non-quantitative MRI analysis. They analyzed a dataset consisting of muscle MRI images with varying degrees of intramuscular fat replacement and edema obtained from 14 patients with facioscapulohumeral dystrophy (FSHD). To calculate the predictive performance of the model, the absolute mean error and the square mean error were used. The feasibility of predicting the FF parameters of quantitative MRI was demonstrated, using texture analysis and machine learning methods from conventional T2W images, which provided mean absolute errors ranging from 0.110 to 0.133 for FF and 0.068 to 0.115 for wT2. The most accurate methods were RF, SVM, and kNN to predict FF, and tree, RF and kNN to predict wT2 [23].

Verdú-Díaz et al analyzed muscle MRI studies of patients with muscular dystrophies. The muscle MRI suggests diagnosis based on the disease-specific patterns of muscle fatty replacement. Thus, the difference among the patterns observed in the muscular dystrophies allows it to identify each dystrophy given its specific features pattern on muscle MRI. RF-supervised ML models were applied and the results were compared with the concepts of four expert radiologists. The analysis included 976 MRI images of pelvic, thigh, and leg muscles of patients with different muscular dystrophies with genetic confirmation. After evaluating 2000 RF models, the final model had a diagnostic accuracy of 95.7%, identifying different types of muscular dystrophy. Comparing the model with reports of radiologists, the accuracy results were 91.6% for the model and 63.3% for radiologists [24].

Gadermayr et al studied muscle MRI studies of 41 participants of whom seven had moderate myopathy characterized by small foci of fat infiltration, 13 had severe myopathies characterized by severe fat infiltration of muscles, and 21 were healthy controls. They used a data gain model (GAN), employed a CNN as a discriminator, and performed muscle segmentation with biomedical data segmentation applications. Applying the Dice similarity coefficient, the results of the analysis were 91% for cases of moderate myopathy, and 88% for cases of severe myopathy [25].

AI in NMD electrodiagnosis

Two of the included studies applied AI models to electromyography (EMG) studies [26, 27]. In Yaman and Subasi's study, they classified and segmented EMG data. Motor unit action potentials (MUAP) data was obtained from 2048 EMG samples from seven patients with myopathy and 13 with neuropathy and seven controls. All measurements of patients and control group were performed by specialist physicians. The technical issues were: impedance of a concentric needle electrode (0.45 mm diameter with a recording surface area of 0.07 mm2; impedance at 20 Hz below 200 kHz) was used to collect EMG signals from the biceps brachii muscle. All signals were collected at 20 kHz for 5 seconds at 12-bit resolution and band-pass-filtered at 5 Hz to 10 kHz. Twenty different MUAPs were acquired from all muscles in the form of five to seven muscle insertions. Needles between the regions were pulled at least 5 mm and the acoustic and visual control of the EMG signal was directed close to the active muscle fibers. In the study, the segmentation of the electromyographic images and classification of MUAPs were performed by applying the functions of wavelets, which are a group of basic functions of a signal that is transformed by Wavelet Packet Decomposition (WPD). The study's authors calculated the wavelet packed coefficients (WPC) for every type of EMG signal, and ensembled classifiers with models of ANN, kNN, SVM, RF, Naive Bayes (NB), and decision trees. And, finally combined and powered the resulting algorithms. The classification accuracy was 97.7% [26].

The study by Nodera et al. extracted the characteristic patterns of six types of electromyographic discharges that were recognized by ML models. They collected both graphic and sound electromyographic recordings and retrieved characteristic features of respective EMG discharges by methods commonly applied to audio research. The files were cut into 2-s segments using the Adobe Audition (Adobe Inc., San Jose, USA) program, yielding up to three files from a single file. To avoid variability of signal intensities on different occasions, the audio root mean square volumes were normalized to −26 decibels. The accuracy of the classification model was 90.4% [27].

AI for detection of amyotrophic lateral sclerosis (ALS) biomarkers

Two studies on ALS biomarkers were included [28, 29]. Greco et al.'s study on biomarker detection in blood samples included 726 blood samples obtained from 41 patients with amyotrophic lateral sclerosis (ALS) and 25 patients with other lower motor neuron diseases (LMND) over 10 years [28]. An SVM** **model was used; 692 blood samples from 35 ALS patients and 20 from LMND patients were used to construct the classification dataset, and the remaining 34 samples of six ALS patients and five LMND patients were used for model evaluation. They made measurements of interleukins, lymphocyte subpopulation count, immunoglobulins A, E, G, and M, cell adhesion factors, cell growth and survival factors, and routine blood biochemistry. The most informative analytes for the entire patient group were the proportion of monocytes, IgM levels, and CD3 lymphocyte count. The model's classification accuracy was 87% for ALS patients and 93% for LMND patients. The study showed the importance of immunological components in motor neuron diseases for discrimination of ALS from other lower motor neuron diseases [28].

Tang et al. studied ALS biomarkers and analyzed ALS patient data from the international open ALS database, including results of different types of blood samples and lung function tests using an unsupervised clustering algorithm. It is striking that when analyzing the accuracy of the model they found that univariate analysis had an accuracy of 70%, while the unsupervised clustering model had 95%. The authors explained the difference by the possibility of doing a multivariate analysis with the unsupervised clustering model [29].

AI in genetic diagnostic studies of NMD

In Tran et al.'s study of identifying gene clusters for different muscle diseases, samples were analyzed from 1260 patients with muscle weakness, of which 824 were cases of myopathy and 436 were controls. They performed an enrichment analysis on groups of genes. The ML model was based on the SVM method. From a total of 34,099 gene expression data, they designed a multiclass classifier based on biological processes. They reported a genetic distinctive consisting of the 500 genes common to all groups. For the specific case of hereditary myopathies, the accuracy of the classification was 90% [30].

Limitations

The present study has limitations. The most important is the heterogeneity of the studies of both the groups of diseases analyzed and the diagnostic methods applied.

## Conclusions

The articles reviewed show that the use of deep learning models for the study and diagnosis of neuromuscular diseases has been increasing. 

The usefulness of AI applications in imaging studies is evidenced in the pre-processing of images through segmentation, and also in the analysis of the characteristics and abnormalities of the tissues studied.

The results that contrast the accuracy of the classification by the different algorithms used are greater than 90% in most studies. This contributes to the accuracy of the diagnosis of patients with NMD. For clinical application, the quality and goodness of algorithms of AI depend on the quality of the data used for the training set and analysis set of data.
